# Effects of vaccination and interventions on nasal microbiome and BRD-associated pathogens in calves

**DOI:** 10.3389/fmicb.2024.1467908

**Published:** 2024-11-18

**Authors:** Guoxing Liu, Sen Zhang, Zhijie Xiang, Ihsanullah Shirani, Yingyu Chen, Aizhen Guo

**Affiliations:** ^1^National Key Laboratory of Agricultural Microbiology, Hubei Hongshan Laboratory, College of Veterinary Medicine, Huazhong Agricultural University, Wuhan, China; ^2^Hubei International Scientific and Technological Cooperation Base of Veterinary Epidemiology, The Cooperative Innovation Center for Sustainable Pig Production, Wuhan, China; ^3^Key Laboratory of Development of Veterinary Diagnostic Products, Ministry of Agriculture and Rural Affair, Wuhan, China

**Keywords:** immune stress, upper respiratory tract, respiratory microbiome, intervention measure, attenuated vaccine

## Abstract

Vaccination is a widely adopted measure to prevent diseases, but the process of immunization can induce a substantial stress response. This study aimed to investigate the impact of a combined *Mycoplasma bovis*-BoHV-1 vaccine on the upper respiratory tract microbiome and BRD-associated pathogens in calves, as well as to evaluate the effects of potential interventions. The results showed that the percentage of *Pasteurella* species in the upper respiratory tract was elevated in calves after vaccination without intervention, and *Pasteurella multocida* was activated and proliferated. Interestingly, none of the three interventions (Sodium selenite-vitamin E, Astragalus polysaccharide and Ceftiofur sodium) affected antibody production after immunization. The administration of sodium selenite-vitamin E and astragalus polysaccharide reduced serum levels of cortisol and malondialdehyde, increased glutathione peroxidase (GSH-Px) and superoxide dismutase (SOD), and alleviated the proliferation of *Pasteurella multocida*. Furthermore, the use of ceftiofur sodium almost completely inhibited the proliferation of *Pasteurella multocida* induced by immune stress. These findings provide a reference for mitigating the negative impacts associated with vaccination and highlight the potential benefits of using targeted nutritional and antimicrobial interventions to optimize immune responses and maintain a stable respiratory microbiome in calves.

## 1 Introduction

Bovine Respiratory Disease (BRD) is a significant concern in the cattle industry, causing substantial economic losses worldwide each year (Bell et al., 2021). BRD is a complex, multifactorial disease involving a combination of bacterial and viral infections.

In healthy cattle, a delicate balance exists between these conditionally pathogenic bacteria and the commensal microbial community of the upper respiratory tract (Chai et al., 2022a; Alexander et al., 2020; Hakansson et al., 2018). The respiratory microbiota and its interactions with the host play a crucial role in cellular function, immune system regulation, and protection against infection by opportunistic pathogens (Zeineldin and Barakat, 2023; Holman et al., 2015). The immune mechanisms of the respiratory tract typically prevent the colonization of pathogens (Zeineldin et al., 2019).

However, certain stressors can induce transient immunosuppression, weakening the immune defenses of the upper respiratory tract (Dhabhar, 2014; Griebel et al., 2014; Holman et al., 2017). This allows pathogenic bacteria and viruses to colonize and replicate, ultimately causing respiratory disease. Vaccination is a widely used strategy to prevent the spread of infectious diseases in livestock. Yet, the process of immunization can trigger a strong stress response in animals, potentially leading to undesirable effects on their health and productivity (Richeson et al., 2019).

Understanding the impact of vaccination on the animal’s physiological processes and microbiome is crucial for developing strategies to mitigate any negative consequences.

The present study aimed to investigate the effect of vaccination with an attenuated and marker *Mycoplasma bovis*-BoHV-1 combined vaccine (attenuated refers to *M. bovis* and the marker BoHV-1 vaccine strain we use is a gene-deleted strain that deletes gG and tk genes) on the upper respiratory tract microbiome and BRD-associated pathogens in calves. To address potential issues, we evaluated the effects of three proposed interventions: sodium selenite-vitamin E (Se-VE), astragalus polysaccharide (APS), and ceftiofur sodium.

By examining the changes in the upper respiratory tract microbiome, the abundance of BRD-associated pathogens, and the antibody response after vaccination, this study provides valuable insights into the complex interactions between the immune system, the animal’s physiology, and the respiratory microbiome. The findings of this research can contribute to the development of more effective strategies to safeguard the health and wellbeing of calves during the vaccination process.

## 2 Materials and methods

### 2.1 Cells and viruses

The wild-type BoHV-1 HB06 strain (GenBank accession number: AJ004801.1) was maintained in the National Key Laboratory of Agricultural Microbiology. Madin-Darby bovine kidney cells (MDBK) were procured from the China Institute of Veterinary Drug Control and used in this study.

### 2.2 Culture of BoHV-1

The BoHV-1 HB06 strain was cultured as previously described (Marawan et al., 2021). Briefly, the BoHV-1 HB06 wild-type strain was previously isolated from clinical samples by our laboratory, and the virus was propagated in Madin-Darby bovine kidney (MDBK) cells using Dulbecco’s modified Eagle’s medium (DMEM) supplemented with 10% fetal bovine serum (Inner Mongolia Opcel Biotechnology Co., Ltd., Hohhot, China). The cell culture was maintained at 37°C in a 5% CO_2_ incubator.

### 2.3 Animal experiments

A total of 15 Holstein dairy calves, aged between two to four months, were included in the study. All calves in our experiment came from the same pasture, and the calves were isolated for a week before the experiment began to minimize the stress of the transfer in the experiment and to ensure the stability of the calve microbiome at the beginning of the experiment. These calves were purchased from pasture and tested seronegative for *M. bovis*, BoHV-1, *Pasteurella* and *Mannheimia haemolytica*. The calves were divided into 5 groups.

All cattle were housed in isolation to prevent cross-infection. The treatment groups were as follows:

1. Sodium selenite-vitamin E (Se/E) group: Immunized with *M. bovis*-BoHV-1 (1.0 × 10^8^ CFU *M. bovis* HB150, 1.0 × 10^6^ TCID_50_ BoHV-1 gG−/tk) combined vaccine administered as nasal drops. Then received an intramuscular injection of sodium selenite-vitamin E (50 g of sodium selenite and 1 g vitamin E per L, 5 mL per calf) on the day of immunization and the third day after immunization.
2. Astragalus polysaccharide (APS) group: Immunized with *M. bovis*-BoHV-1 combined vaccine administered as nasal drops. Then received an intramuscular injection of astragalus polysaccharide (0.01 g/mL, 0.2 mL/kg) on the day of immunization and the first three day after immunization.
3. Ceftiofur sodium (CS) group: Immunized with *M. bovis*-BoHV-1 combined vaccine administered as nasal drops. Then received an intramuscular injection of ceftiofur sodium (1.7 mg/kg per day) for the first three day after immunization.
4. Vaccine Control (VC) group: Immunized with *M. bovis*-BoHV-1 combined vaccine administered as nasal drops, and did not receive any additional treatment.
5. Mock group: Did not receive any treatment or vaccination.

### 2.4 Clinical evaluation and sample collection

The calves nostrils are cleaned and sterilized before we collect the nasal swab samples. The swabs we use are individually wrapped and sterile, and we collect the samples to ensure that the swab goes 15–20 cm deep into the calves nasal cavity. Nasal drops and no additional treatment samples were collected daily for 28 days following immunization. The samples were stored at −80°C for subsequent quantitative PCR (qPCR) analysis and 16S rRNA gene sequencing.

Procoagulant tubes contains thrombin and cabosil were used to collect blood samples from the jugular vein. Blood samples were collected weekly throughout the duration of the experiment. On top of that, blood samples were also collected on days 1, 3, and 5 after immunization for additional analysis.

### 2.5 16S rRNA gene sequencing and bioinformatics analysis

Microbial genomic DNA was extracted from nasal swabs collected on the day of immunization and on the 7th day after immunization. The DNA extraction was performed using the E.Z.N.A.® Tissue DNA Kit (Omega Bio-tek, Norcross, GA, U.S.) according to manufacturer’s instructions. The quality and concentration of the extracted DNA were assessed by 1.0% agarose gel electrophoresis and a NanoDrop2000 spectrophotometer (Thermo Scientific, United States).

The hypervariable region V3-V4 of the bacterial 16 s rRNA gene were amplified with primer pairs 338F (5’-ACTCCTACGGGAGGCAGCAG-3′) and 806R (5’-GGACTACHVGGGTWTCTAAT-3′) in a T100 Thermal Cycler PCR thermocycler (BIO-RAD, USA). The PCR product was extracted from a 2% agarose gel and purified using the PCR Clean-Up Kit (YuHua, Shanghai, China) according to the manufacturer’s instructions. The purified amplicons were quantified using Qubit 4.0 fluorometer (Thermo Fisher Scientific, USA).

The purified amplicons were pooled in equimolar amounts and subjected to paired-end sequenced on an Illumina PE300/PE250 platform (Illumina, San Diego, USA) according to the standard protocols by Majorbio Bio-Pharm Technology Co. Ltd. (Shanghai, China).

Raw FASTQ files from the sequencing data were de-multiplexed using an in-house Perl script. The sequences were then quality-filtered using fastp version 0.19.6 and merged using FLASH version 1.2.7.

The optimized sequences were clustered into operational taxonomic units (OTUs) using UPARSE 7.1 with a 97% sequence similarity level. The most abundant sequence for each OTU was selected as a representative sequence.

To minimize the effects of sequencing depth on alpha and beta diversity measure, the number of 16S rRNA gene sequences from each sample were rarefied to 56,182, which still yielded an average Good’s coverage of 99.75%.

The taxonomy of each OTU representative sequence was analyzed using the RDP Classifier version 2.2 against the 16S rRNA gene database (eg. Silva v138) with a confidence threshold of 0.7.

Alpha diversity indices, such as observed OTUs, Chao1 richness, Shannon’s index, and Good’s coverage, were calculated using Mothur v1.30.1 based on OTUs information.

### 2.6 Shedding of BRD-associated pathogenic bacteria

qPCR was used to quantify the abundance of the following BRD-associated pathogenic bacteria in the collected nasal swab samples: *Pasteurella multocida, Mannheimia haemolytica, Histophilus somni, Trueperella pyogenes* and *Mycoplasma bovis* (wild-type strain). The primers and probes used for the qPCR analysis are detailed in Table 1.

**Table 1:** Primers and probes used in this study.

Target pathogen	Target gene	Sequence (5′-3′)	Gene length
*Pasteurella multocida*	kmt-1	Forward: GGGCTTGTCGGTAGTCTTT Reverse: CGGCAAATAACAATAAGCTGAGTA Probe: FAM-CGGCGCAACTGATTGGACGTTATT-TAMRA	148 bp
*Mannheimia haemolytica*	1sodA	Forward: ATTAGTGGGTTGTCCTGGTTAG Reverse: GCGTGATTTCGGTTCAGTTG Probe: FAM-CTGAACCAACACGAGTAGTCGCTGC-TAMRA	144 bp
*Histophillus somni*	16S-rRNA F	Forward: AAGGCCTTCGGGTTGTAAAG Reverse: CCGGTGCTTCTTCTGTGATTAT Probe: FAM-CGGTGATGAGGAAGGCGATTAG-TAMRA	93 bp
*Trueperella pyogenes*	plo-Pyolysin	Forward: ATCAACAATCCCACGAAGAG Reverse: TTGCAGCATGGTCAGGATAC Probe: FAM-TCGACGGTTGGATTCAGCGCAATA-TAMRA	99 bp
*Mycoplasma bovis*	Mbovp0732	Forward: AGCGACCAAAATACTAGAC Reverse: TCGTTGCCACTGTATTCA	140 bp

### 2.7 Serum antibody response to *Mycoplasma bovis*

Serum antibodies against *M. bovis* were detected using a competitive enzyme-linked immunosorbent assay (ELISA) as previously described (Zhang et al., 2024). Briefly, the test serum samples were diluted fourfold, and then added along with HRP-labeled monoclonal antibodies to an *M. bovis* p579 protein-coated plate. Positive and negative serum controls were also included. The plate was incubated at 37°C for 60 min. After washing, 100 μL of substrate chromogenic solution was added to each well and incubated at room temperature, away from light, for 10 min. The OD_450nm_ value was immediately read after stopping the reaction.

The blocking rate (PI value) was calculated as follows:

Blocking rate = 1 - (Sample OD_450nm_/Mean OD_450nm_ of negative control serum).

Conditions for the establishing the test:

0.65 < OD_450nm_ negative control <2.0.

PI value of positive control >0.6.

PI value of sample ≥ 0.41 indicates a positive result.

PI value of sample < 0.41 indicates a negative result.

### 2.8 Virus neutralization assay

Serum samples were first heat-inactivated at 56°C for 30 min. The inactivated serum was then serially diluted in a 96-well cell culture plate. Next, 100 TCID_50_ of BoHV-1 HB06 virus was incubated with the diluted serum samples at 37°C in a 5% CO_2_ incubator for 1 h. This allowed any neutralizing antibodies in the serum to bind to the virus. The serum-virus mixture was then transferred to a 96-well cell culture plate containing MDBK cells. The plate was cultured in a 5% CO_2_ incubator at 37°C for three days. The Neutralizing antibody titers were calculated using the Reed-Muench method. This method determines the highest serum dilutions that successfully inhibits BoHV-1 infection of the MDBK cells.

### 2.9 Serum biomarker measurement

Serum levels of Cortisol (COR) superoxide dismutase (SOD), glutathione peroxidase (GSH-Px) and malondialdehyde (MDA) were measured using commercial kits. Specifically, serum COR levels were measured using commercial ELISA kits (Meimian Industrial Co., Ltd. Jiangsu, China), while serum SOD, GSH-Px and MDA levels were measured using kits from Beyotime Biotechnology (Shanghai, China). All measurements were performed according to the manufacturer’s instructions.

### 2.10 Ethics statement

The animal experiment protocol was approved by the Animal Experiment Ethics Committee of Huazhong Agricultural University (Huazhong Agricultural University Ethics Approval Number: HZAUCA-2024-0036) and conducted in strict accordance with the Guidelines for the Care and Use of Laboratory Animals of Wuhan, Hubei, China.

### 2.11 Statistical analysis

Shapiro–Wilk normality test was used for normal distribution. Statistical analysis was performed using Student’s t-test and one-way analysis variance (ANOVA). Significant differences between groups were determined using the following *p*-value thresholds: *p* < 0.05 (*), *p* < 0.01 (**), *p* < 0.001 (***), or *p* < 0.0001 (****). Error bars in the figures represented the standard error of the mean.

## 3 Results

### 3.1 Analysis of microbiome diversity

To assessed microbiome diversity, 16S rRNA gene sequencing was used. The results showed that after vaccination, the Se/E, APS, CS, VC and Mock group had 844, 1,311, 931, 925 and 1,523 operational taxonomic units (OTUs), respectively.

**Figure 1:**
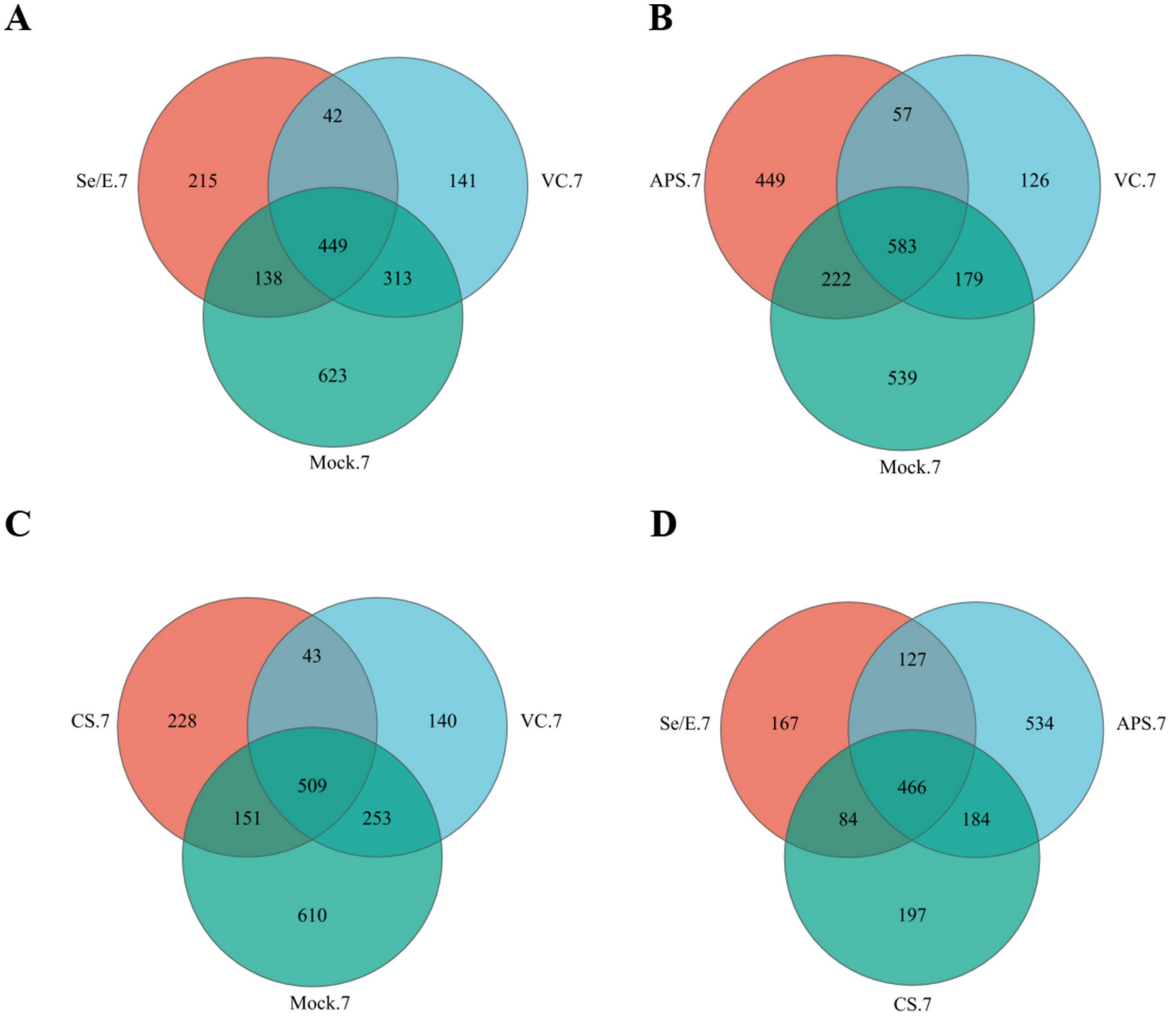
Venn diagrams represent the shared and exclusive OTUs of upper respiratory tract bacterial communities at 97% similar levels on the 7 day after immunization. In (A) Se/E, VC, and Mock groups, (B) APS, VC, and Mock groups, (C) CS, VC, and Mock groups, (D) Se/E, APS, and CS groups.

In the Se/E group, 69.55% (587/844) of the OTUs were shared with the Mock group, and 58.18% (491/844) were shared with the VC group. Of these, 53.20% (449/844) were present in all three groups (Figure 1A).

In the APS group, 61.40% (805/1311) of the OTUs were shared with the Mock group, and 48.82% (640/1311) were shared with the VC. Of these, 44.47% (583/1311) were present in all three groups (Figure 1B).

In the CS group, 70.89% (660/931) of the OTUs were shared with the Mock group, and 59.29% (552/931) were shared with the VC group. Of these, 54.67% (509/931) were present in all three groups (Figure 1C).

For the Se/E, APS and CS groups, 466 OTUs were shared, which occupied 55.21% (466/844) of the Se/E group, 35.55% (466/1311) of the APS group and 50.05% (466/931) of the CS group (Figure 1D).

**Figure 2:**
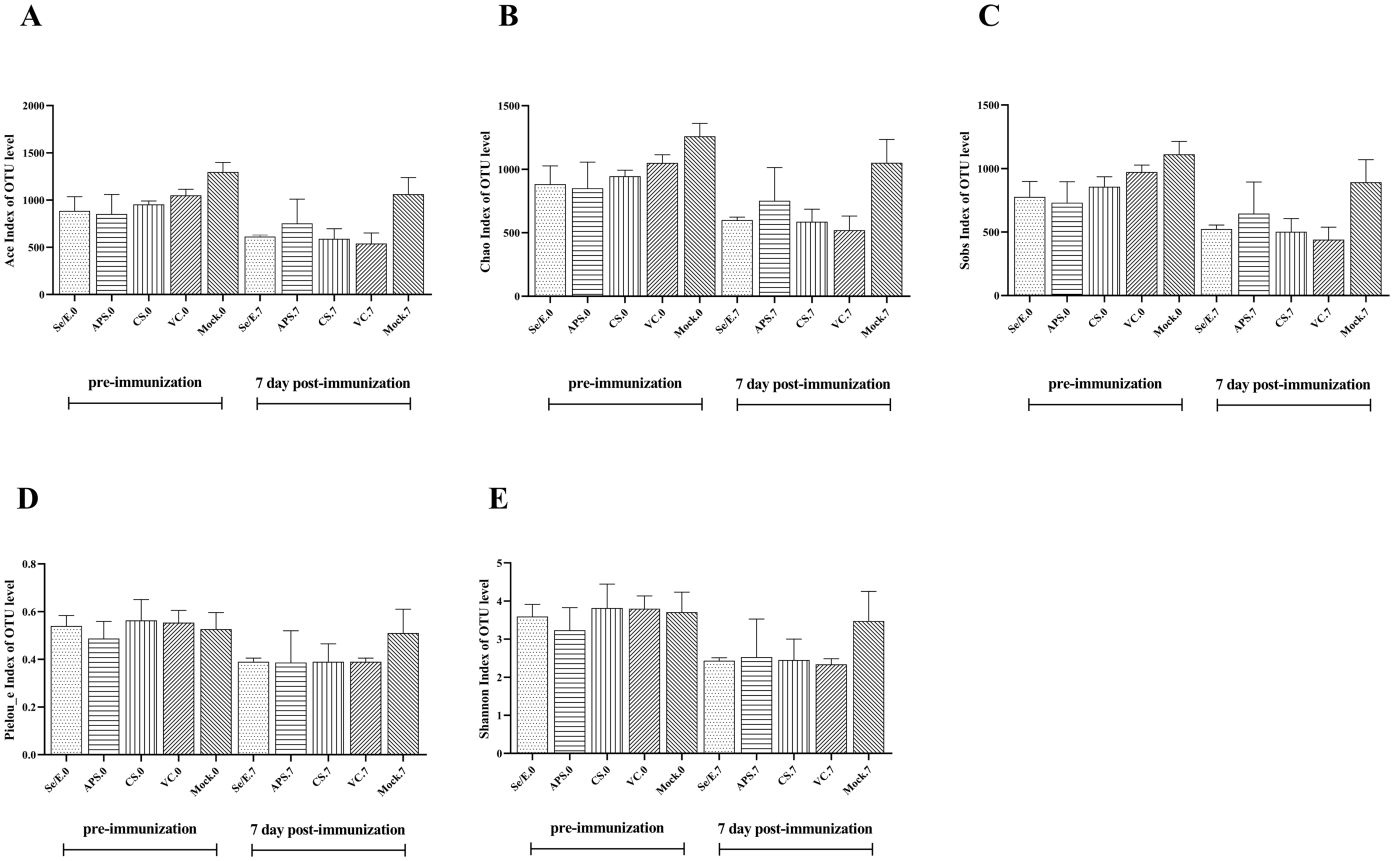
Pre- and post-immunization alpha diversity indices include (A) the ACE index, (B) the Chao index, (C) the Sobs index, (D) the Pieuo_e index and (E) the shannon index. Each horizontal coordinate unit represents one group.

Additionally, ACE, Chao, Sobs, Pielou_e, and Shannon index were used to evaluate the microbiome diversity pre-immunization (day 0) and post-immunization (day 7) (Figures 2A–E). The data showed no statistically significantly difference in these diversity measures among all the treatment groups at both time points, day 0 and day 7. However, the Chao, Sob, Ace indices indicated that microbiome diversity decreased by nearly 50% on day 7 compared to day 0 across the Se/E, APS, CS and only vaccine groups, though this reduction was not statistically significant.

These findings suggested that while the immunization and various supplemental treatments did not significantly alter the overall microbiome diversity, there was a general trend toward reduced diversity approximately one week after immunization, regardless of the treatment group. The lack of statistical significance implies the diversity changes were modest and not drastically impacted by the different interventions.

### 3.2 Top ten most abundant bacterial phyla in the nares

**Figure 3:**
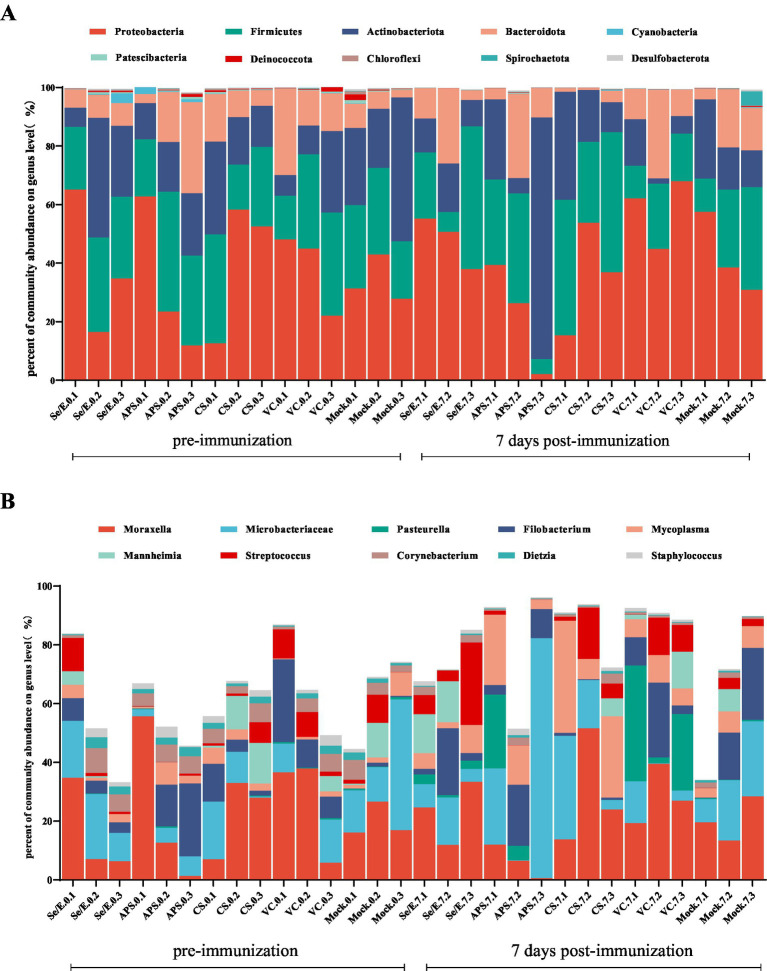
Bar graphs show the most abundant ten bacterial groups in the nares at the (A) phylum level and (B) genus level. Each horizontal coordinate unit represents one cattle; as an example, Se/E.0.1 represents the first cattle in the Se/E group on the day of immunization, and Se/E.7.1 represents the first cattle in the Se/E group on the 7 day after immunization.

To further analyze the species composition, the study examined the abundance of the top ten bacteria in the upper respiratory tract. At this level, a total of 32 phyla were identified across all samples, with the dominant phyla being *Proteobacteria* (39.16% on average), *Firmicutes* (26.89%), *Actinobacteriota* (20.64%) and *Bacteroidota* (11.67), which together accounted for 93.01 to 99.68% of the total (Figure 3A).

At the genus level, a total of 1,003 genera were identified in all samples. The top ten genera in terms of abundance were *Moraxella* (21.72%), *Microbacteriaceae* (15.13%), *Pasteurella* (3.65%), *Filobacterium* (8.75%), *Mycoplasma* (7.12%), *Mannheimia* (3.47%), *Streptococcus* (4.84%), *Corynebacterium* (3.01%), *Dietzia* (1.15%) and *Staphylococcus* (1.14%) (Figure 3B).

**Figure 4:**
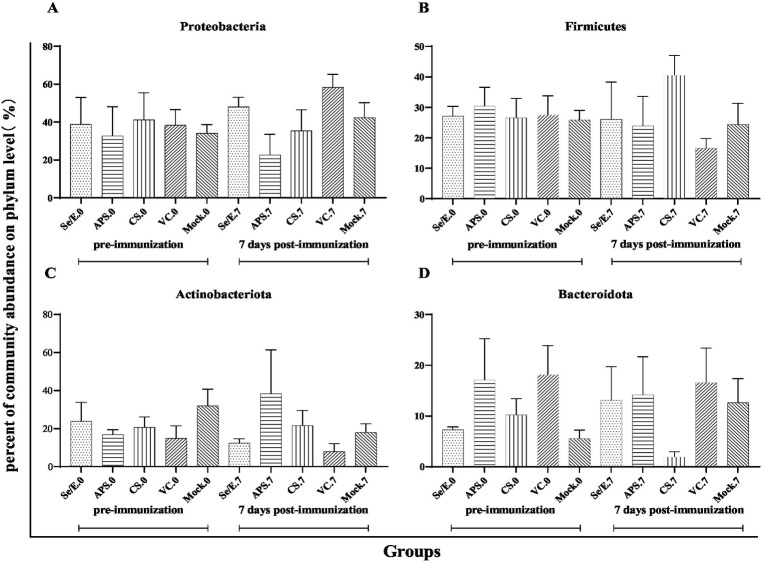
Differences in relative abundance of dominant phylum before and after immunization and between groups after immunization. (A) *Proteobacteria*, (B) *Firmicutes*, (C) *Actinobacteriota* and (D) *Bacteroidota*.

**Figure 5:**
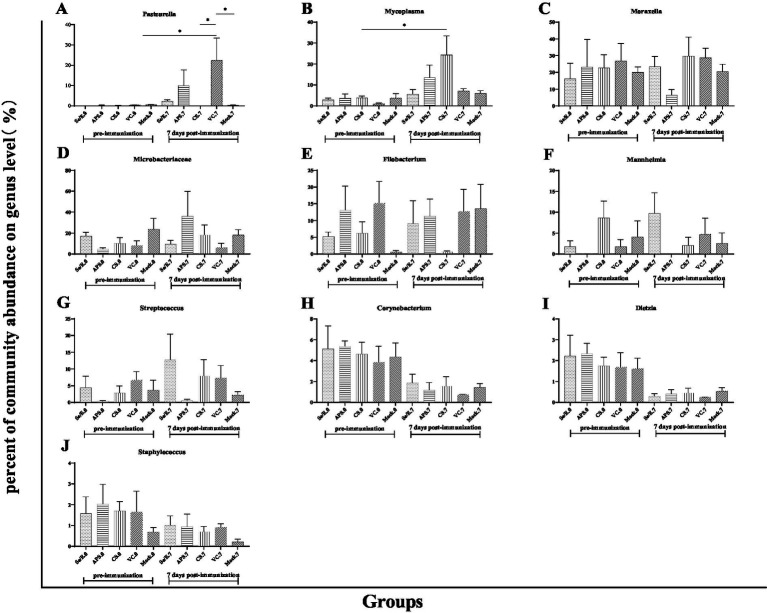
Differences in the relative abundance of the top ten genera before and after immunization and between groups after immunization. (A) *Pasteurella*, (B) *Mycoplasma*, (C) *Moraxella*, (D) *Microbacteriaceae*, (E) *Filobacterium*, (F) *Mannheimia*, (G) *Streptococcus*, (H) *Corynebacterium*, (I) *Dietzia* and (J) *Staphylococcus.* The symbol * means *p* < 0.05 and there exist different the groups.

Further analysis showed that the above four dominant phyla did not change significantly before and after immunization across all groups (Figures 4A–D). However, on day 7 post-immunization, the abundance of *Pasteurella* significantly increased in the VC group compared to day 0 (*p* < 0.05), and was also significantly higher than the CS (*p* < 0.05) and Mock groups (*p* < 0.05) (Figure 5A). Meanwhile, on day 7 post-immunization, the abundance of *Mycoplasma* in the CS group was significantly increased compared to the pre-immunization period (*p* < 0.05), and also showed a notable increase compared to all other groups (Figure 5B). Other pathogens showed no significantly difference (Figures 5C–J).

These results indicate that while the overall microbial community structure at the phylum level remained relatively stable, there were some significant shifts in the abundance of specific genera, such as *Pasteurella* and *Mycoplasma*, in response to the different immunization and supplementation treatments.

### 3.3 Detection of BRD-associated pathogens

**Figure 6:**
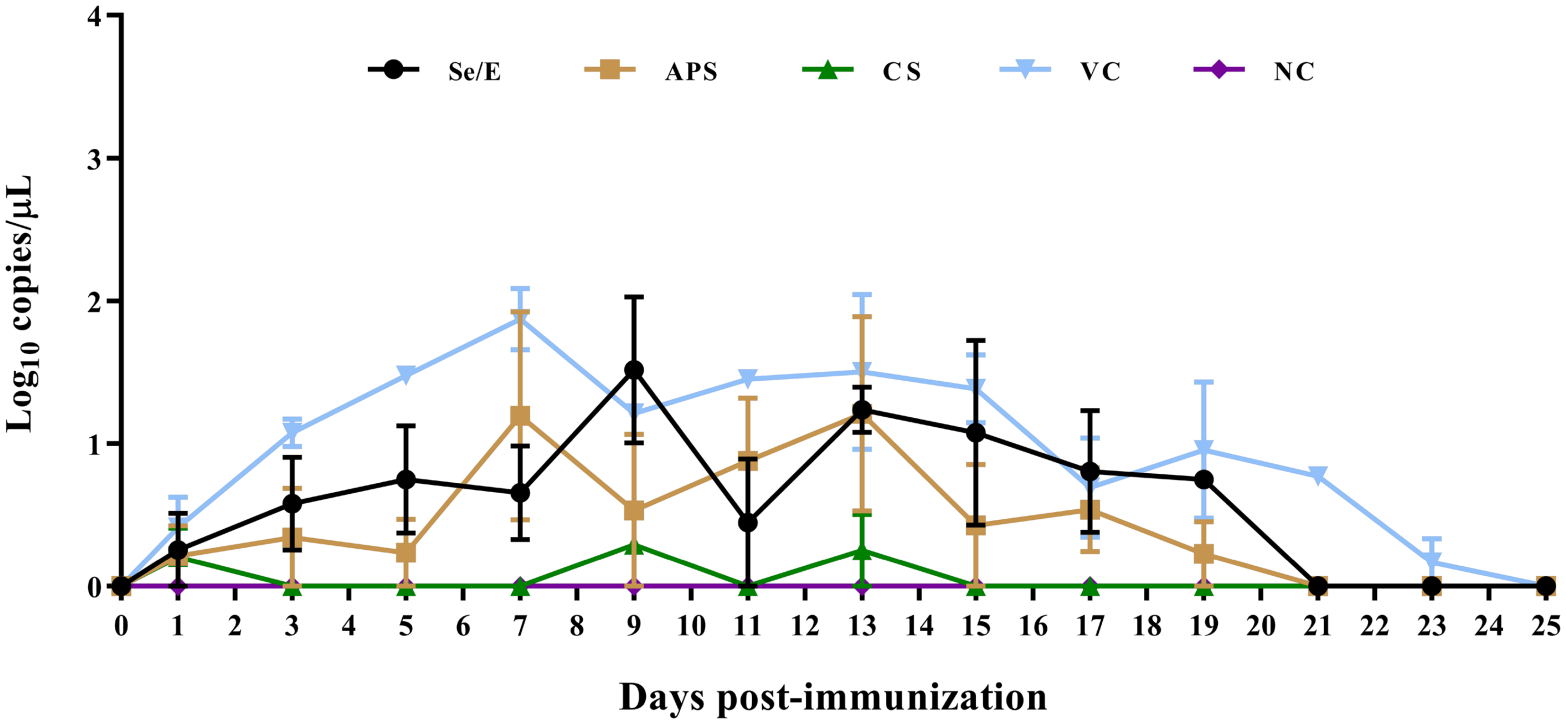
Detection of *Pasteurella multocida* shedding in nasal swabs after immunization.

To evaluate the impact of the different treatment regimens on common respiratory pathogens in the calves post-immunization, we detected the shedding of *Pasteurella multocida*, *Mannheimia haemolytica*, *Histophilus somni*, *Trueperella pyogenes*, and *Mycoplasma bovis*. However, only *Pasteurella multocida* was successfully detected (Figure 6).

Among all groups, the animals that were vaccinated only with the *M. bovis*-BoHV-1 vaccine, without any additional treatment, had the highest level of *Pasteurella multocida* shedding throughout the observation period. On 7 day post immunization, the *Pasteurella multocida* shedding reached a peak of 10^1.87^ copies/μL. Thereafter, the shedding gradually decreased, and by 25 days post-immunization, no *Pasteurella multocida* shedding was detected in this group.

Se/E group had a lower *Pasteurella multocida* shedding compared to the vaccinated only group. The shedding in the Se/E group increased to 10^1.52^ copies/μL on day 9 post-immunization. It then decreased to 10^0.45^ on day 11, and subsequently returned to 10^1.21^ copies/μL. After that, the *Pasteurella multocida* shedding in the Se/E group gradually decreased until day 21 days post-immunization.

The APS group had lower *Pasteurella multocida* shedding than the Se/E group in the first 5 days post-immunization than. However, the APS group reached a peak of 10^1.2^ copies/μL on day 7, two days earlier than the Se/E group. The shedding then decreased to 10^0.53^, similar to the Se/E group. But the APS group experienced an increase in shedding again from day 9 to 13, before gradually decreasing. By day 21, no *Pasteurella multocida* shedding was detected in the APS group, similar to the Se/E group.

The CS group exhibited the lowest *Pasteurella multocida* shedding among all the treatment groups. *Pasteurella multocida* shedding was only detected on day 1, 9 and 13, with the levels of 10^0.2^, 10^0.29^, and 10^0.25^ copies/μL, respectively. On all other days, no *Pasteurella multocida* shedding was detected in the CS group.

These indicated that the addition of all the three supplemental treatments can help reduce the level of *Pasteurella multocida* shedding in calves compared to the group that was vaccinated only without any other treatment. The antibiotic treatment with CS was the most effective in minimizing *Pasteurella multocida* shedding, with only sporadic and low-level detection in the CS group compared to the other treatment groups. The timing of the peak *Pasteurella multocida* shedding varied between the groups, the APS group peaking the earliest ahead of the other treatment groups.

### 3.4 Changes in blood parameters

**Figure 7:**
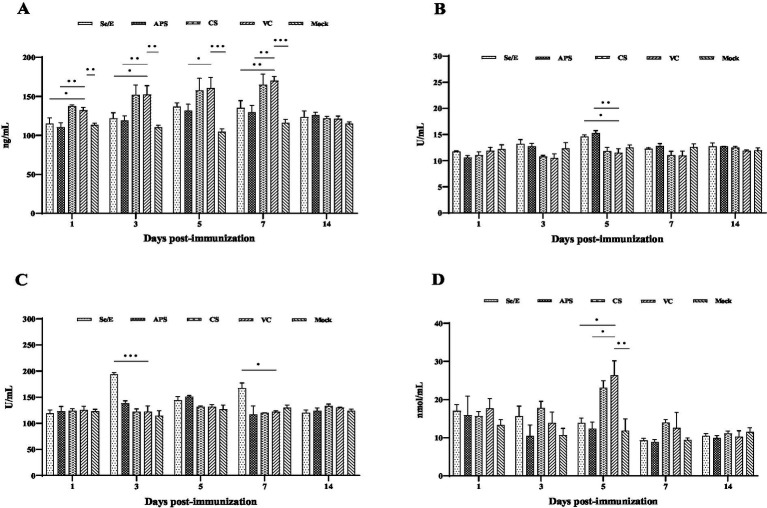
Effect of intervening drugs on serum levels of (A) COR, (B) SOD, (C) GSH-Px and (D) MDA in calves at 1, 3, 5, 7 and 14 day after vaccination. Variation is expressed as standard deviation. The symbol *, ** and *** means *p* < 0.05, *p* < 0.01 and *p* < 0.001, respectively, and there exist significantly different the groups.

For the COR measurement, the Se/E and APS groups showed almost the same COR levels compared to the Mock group throughout the entire observation period. The CS and VC groups did not differ significantly from each other, but both had extremely higher COR levels compared to the other three groups from day 1 to 7. On day 14, all groups had the same COR level (Figure 7A).

For the SOD measurement, all groups maintained similar levels without significant differences, except on day 5. On day 5, the Se/E and APS groups exhibited a significantly higher SOD levels compared to the VC group (Figure 7B).

For the GSH-Px measurement, the Se/E groups exhibited an extremely significantly higher level compared to VC group on day 3 and 7. In contrast, the other groups maintained relatively stable levels throughout the whole observation, without any significant differences among them (Figure 7C).

For the MDA measurement, all groups maintained similar levels, except on day 5. On day 5, the VC groups exhibited a significantly higher level compared to the Se/E and Mock groups. Additionally, while not statistically significant, the CS had a notable higher MDA level compared to the Se/E, APS and Mock groups, especially on day 5 (Figure 7D).

These results indicated that the Se/E and, to a lesser extent, the APS groups were able to better preserve the antioxidant status and regulate the stress response. And CS intervention was not effective in mitigating the negative impacts of the stressors experienced by the animals.

### 3.5 Antibody response

#### 3.5.1 *Mycoplasma bovis* serum antibody levels

**Figure 8:**
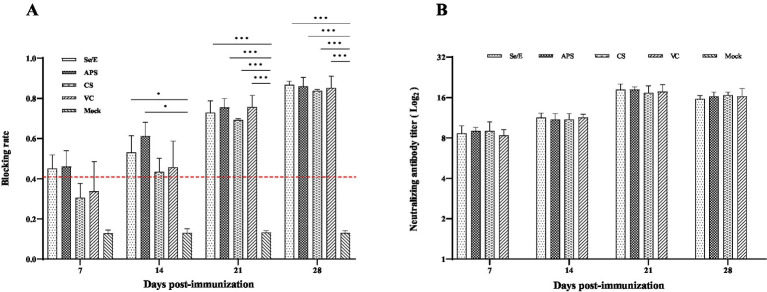
Humoral immune responses induced by the *M. bovis*–BoHV-1 combined vaccine. Serum samples were collected weekly to determine (A) serum antibody against *M. bovis* and (B) BoHV-1 serum neutralizing antibody. Variation is expressed as standard deviation. The red line represents the negative–positive threshold of *M. bovis* antibody. The symbol *, ** and *** means *p* < 0.05, *p* < 0.01 and *p* < 0.001, respectively, and there exist significantly different the groups.

*Mycoplasma bovis* serum antibody levels were detected by competitive ELISA. During the entire observation period, all vaccine groups exhibited notably higher antibody levels compared to the Mock control group. Especially on day 21 and 28, the vaccine groups (Se/E, APS, CS and VC) showed statistically significantly higher antibody levels. The Se/E and APS groups, in particular, demonstrated consistently significantly higher antibody levels from day 14 to day 28 compared to the Mock control group (Figure 8A).

#### 3.5.2 BoHV-1-neutralizing antibody response

Serum neutralization assay was used to determine the level of BoHV -1 neutralizing antibody in calves. Except for the Mock group, all other treatment groups exhibited high levels of neutralizing antibodies starting from day 7 post-immunization. At 21 days post-immunization, all vaccined groups reached the peak of their BoHV -1 neutralizing antibody response. The vaccine groups maintained these elevated neutralizing antibody titers through the remainder of the observation period (Figure 8B).

## 4 Discussion

Evidence suggests that the respiratory microbiota plays an important role in determining respiratory health and preventing colonization by respiratory pathogens (Klima et al., 2019; Timsit et al., 2016a; Timsit et al., 2016b; Man et al., 2017; Amat et al., 2023; Crosby et al., 2022; Timsit et al., 2020). The respiratory tract microbiome of calves starts to colonize shortly after birth and is subsequently affected by a variety of factors, such as age, diet, season, and production management strategies (Zeineldin et al., 2019). Stress is inevitable in production and it can adversely affect the production of animals. In addition, stress can also cause changes in the respiratory microbiome (Malmuthuge et al., 2021). Studies have shown that the bacterial flora of the respiratory tract can be affected by transport, especially long-distance transport, which may increase the relative abundance of BRD pathogens (Chai et al., 2022b). In this study, we evaluated the impact of different supplemental treatments (Se/E, APS, and CS) on the microbiome and immune responses of calves following immunization against *M. bovis* and BoHV-1. The results indicated while the overall microbiome diversity was not significantly altered by the immunization and supplemental treatments, there was a general trend toward reduced diversity around one week post-immunization across all groups. This suggested the immunization process itself may have a modest impact on the calf microbiome, regardless of the supplemental treatments provided (shown in Figure 2).

At the genus level, the microbiome was dominated by commensal and potentially pathogenic bacteria, including *Moraxella*, *Microbacteriaceae*, *Pasteurella*, *Filobacterium*, *Mycoplasma*, *Mannheimia*, and *Streptococcus*. Notably, the abundance of *Pasteurella* and *Mycoplasma* increased significantly in the vaccinated-only (VC) group compared to other treatment groups on day 7 post-immunization. This indicates the supplemental treatments, particularly the antibiotic CS, were effective in mitigating the overgrowth of these potentially pathogenic genera following immunization (shown in Figure 5).

*Pasteurella multocida* is a major causative agent of BRD, often inducing severe mortality in combination with other pathogens. Using qPCR, we tracked the abundance of bacterial pathogens associated with BRD after immunization with combined vaccine, we found that *Pasteurella multocida* could be detected in nasal swabs and maintained for a period following immunization.

The appearance of *Pasteurella multocida* after vaccination may be due to calves being in a state of chronic stress, with their immune systems suppressed to a certain extent, limiting their ability to inhibit the proliferation of conditionally pathogenic bacteria like *Pasteurella multocida*. The source of these bacteria could be from the external environment, as interactions between cattle and their surroundings can contribute to the upper respiratory microbiota. Cattle, especially calves, frequently lick their environment and each other, providing opportunities for environmental bacteria to reach the upper respiratory tract (Chai et al., 2022a).

The use of sodium selenite-VE and astragalus polysaccharides moderated the proliferation of these conditionally pathogenic bacteria associated with immune stress, in terms of both the duration and abundance detected in nasal swabs. Notably, the antibiotic ceftiofur sodium was most effective in minimizing *Pasteurella multocida* shedding, with only sporadic and low-level detection compared to the other treatment groups. Interestingly, the timing of the peak *Pasteurella multocida* shedding also differed between groups, with the astragalus polysaccharide group peaking earliest. This suggested the supplemental treatments might have varying effects on the kinetics of pathogen shedding, a critical finding (shown in Figure 6).

Gut microbiome are essential for human development and function, especially for the initiation and maturation of the adaptive immune system. The use of antibiotics leads to the reduction of intestinal microbial diversity, changes in metabolic activity, and the generation of Antibiotic-resistant microorganisms, which can lead to the development of diseases (Ramirez et al., 2020; Vasco et al., 2023). In addition, reports have shown that calves are an important source of antibiotic-resistant gene transmission on dairy farms and that there is a close association between Apart from antimicrobial usage and antibiotic-resistance (Ferroni et al., 2022; Salerno et al., 2022). In this study, we found significant changes in the proportion of abundance of the genera except *Pasteurella* and *Mycoplasma* after ceftiofur sodium treatment. Other genera, which also contained some non-pathogenic bacteria, did not show significant changes in abundance ratio. Considered the over antimicrobial resistance and disruption of the gut microbiome, although ceftiofur sodium was effective in minimizing *Pasteurella multocida* shedding, the use of antibiotics in calves is not recommended.

Stress can directly or indirectly activate the hypothalamic–pituitary–adrenal axis, leading to the secretion and release of glucocorticoids (primarily cortisol) from the adrenal zona fasciculata (Bellavance and Rivest, 2014). This ultimately increases the concentration of cortisol in the blood. The increase in blood cortisol causes immunological changes that produce inflammatory response, which in turn increases the production of reactive oxygen species and leads to oxidative stress (Jung et al., 2023). Oxidative stress is caused by an imbalance between pro-oxidants and antioxidants in the organism.

In the current study, we found that ceftiofur sodium can induce higher cortisol and malondialdehyde levels. This can have far-reaching consequences, including oxidative stress, metabolic dysregulation, immune system impairment, neurological and psychological effects, and cardiovascular complications (Jung et al., 2023; Knezevic et al., 2023; Aresta et al., 2021; Dhabhar, 2009; Baudrand and Vaidya, 2015). Understanding these pathways is crucial for identifying potential interventions to mitigate the negative impacts of chronic ceftiofur sodium exposure.

Astragalus polysaccharide is a key active ingredient isolated from Astragalus membranaceus and has received much attention due to its anti-inflammatory, anti-oxidative stress, and immunomodulatory functions (Qi et al., 2017; Chen et al., 2020). Se is essential for organisms and participates in redox reactions, while Vitamin E acts as an antioxidant and enhances Se absorption (Zheng et al., 2021). Therefore, Se and vitamin E are often used to reduce the effects of oxidative stress. Ceftiofur sodium, as a common clinical treatment for BRD, inhibits BRD-associated pathogens but has no effect on *M. bovis*, one of the components of the combined vaccine. In the present study, we investigated the intervention effects of these three components on the negative effects of stress caused by vaccination.

Our results shown that the COR levels in Se/E and APS groups were comparable to the Mock group on day 3 post-immunization, and not statistically different from the Mock group, despite experiencing a slight elevation. This suggested that the use of Se-VE and APS may alleviate the stress caused by immunization.

The main antioxidants in organisms are GSH-Px, SOD and catalase (CAT). Oxidative stress manifests in the accumulation of reactive oxygen species (ROS) and MDA, as well as a decrease in the expression or activity of enzymes, ultimately causing cellular and tissue damage (Zhu et al., 2018; Samarghandian et al., 2017). Only on day 5 after immunization, SOD in Se/E and APS groups was significantly higher than the Mock group, and MDA was lower. The concentration of GSH-Px in Se/E group was significantly higher than the other groups on day 3 and 7 after immunization. These results demonstrated that the use of Se-VE and APS can reduce the effects of oxidative stress to a certain extent (shown in Figure 7).

Although there was no significant difference between the immunized groups in either serum antibody against *M. bovis* or BoHV-1 neutralizing antibodies after immunization, serum antibody against *M. bovis* appeared to elevate faster in the Se/E and APS groups. This suggested that the three interventions did not affect the immunization effect of the vaccine, but the use of Se-VE and APS seemed to accelerate the production of antibodies (shown in Figure 8).

Interestingly, the abundance percentage of *Mycoplasma* in the CS group increased rapidly on day 7 after immunization. We speculated that this might be related to the slow rise in *M. bovis* antibody levels in the CS group compared to the Se/E and APS groups. This indicates that the use of CS may have had a negative impact on the animals’ immune responses to the vaccine, potentially by disrupting the mucosal microbiome or other mechanisms. In contrast, the Se/VE and APS interventions appeared to support a more robust and timely antibody response to the *M. bovis* component of the vaccine, which could translate to improved protection against Mycoplasma infections in the calves. These findings highlight the importance of considering the impacts of antimicrobial and supportive therapies on vaccine efficacy and the overall immune competence of the animals.

As mentioned above, immune stress increased the relative abundance of *Pasteurella* species in the upper respiratory tract of calves and can cause proliferation of *Pasteurella multocida*. The use of Se-VE and APS after vaccination was found to effectively alleviate the stress response brought about by vaccination, improve the calves antioxidative stress ability, and mitigate the activation and proliferation of *Pasteurella multocida* induced by immune stress, without adversely affecting antibody production.

Due to the limitation of the number of experimental animals, further study is still needed to fully assess vaccination-induced immune stress on the upper respiratory tract microbiome of calves and confirm the benefits of the Se-VE and APS interventions. However, these findings highlight the potential advantages of using appropriate nutritional and anti-inflammatory supplements in BRD vaccination programs to optimize immune responses and maintain a stable respiratory microbial community.

The results provide a basis for further investigation into the role of nutritional supplementation in enhancing vaccine efficacy and preventing respiratory disease outbreaks in cattle. Modulating the immune response and respiratory microbiome through targeted interventions could be a promising approach to improve the overall health and productivity of vaccinated calves.

## 5 Conclusion

This study demonstrated that the addition of Sodium selenite-vitamin E, Astragalus polysaccharide, and ceftiofur sodium (CS) supplements to vaccination regimens helped attenuate the overgrowth of potential pathogenic genera like *Pasteurella* following immunization, with better outcomes compared to vaccination alone. Furthermore, Se/E and APS supplements appeared to better support the development of protective antibody responses against *M. bovis* and BoHV-1.

## Data Availability

We have confirmed that our data is publicly available. And the data are openly available in “science data bank” (CSTR: https://cstr.cn/31253.11.sciencedb.16125; DOI: https://doi.org/10.57760/sciencedb.16125).
